# The impact of heme biosynthesis regulation on glioma aggressiveness: Correlations with diagnostic molecular markers

**DOI:** 10.3389/fnmol.2022.928355

**Published:** 2022-09-14

**Authors:** Mario Mischkulnig, Barbara Kiesel, Thomas Rötzer-Pejrimovsky, Martin Borkovec, Alexandra Lang, Matthias Millesi, Lisa I. Wadiura, Shawn Hervey-Jumper, Josef M. Penninger, Mitchel S. Berger, Georg Widhalm, Friedrich Erhart

**Affiliations:** ^1^Department of Neurosurgery, Medical University of Vienna, Vienna, Austria; ^2^Central Nervous System Tumors Unit, Comprehensive Cancer Center, Medical University of Vienna, Vienna, Austria; ^3^Division of Neuropathology and Neurochemistry, Department of Neurology, Medical University of Vienna, Vienna, Austria; ^4^Department of Statistics, Ludwig-Maximilians-University of Munich, Munich, Germany; ^5^Department of Neurological Surgery, University of California, San Francisco, San Francisco, CA, United States; ^6^Institute of Molecular Biotechnology of the Austrian Academy of Sciences, Vienna, Austria; ^7^Department of Medical Genetics, Life Sciences Institute, University of British Columbia, Vancouver, BC, Canada

**Keywords:** heme biosynthesis, glioma, molecular markers, mRNA, TCGA

## Abstract

**Background:**

The prognosis of diffusely infiltrating glioma patients is dismal but varies greatly between individuals. While characterization of gliomas primarily relied on histopathological features, molecular markers increasingly gained importance and play a key role in the recently published 5*^th^* edition of the World Health Organization (WHO) classification. Heme biosynthesis represents a crucial pathway due to its paramount importance in oxygen transport, energy production and drug metabolism. Recently, we described a “heme biosynthesis mRNA expression signature” that correlates with histopathological glioma grade and survival. The aim of the current study was to correlate this heme biosynthesis mRNA expression signature with diagnostic molecular markers and investigate its continued prognostic relevance.

**Materials and methods:**

In this study, patient data were derived from the “The Cancer Genome Atlas” (TCGA) lower-grade glioma and glioblastoma cohorts. We identified diffusely infiltrating gliomas correlating molecular tumor diagnosis according to the most recent WHO classification with heme biosynthesis mRNA expression. The following molecular markers were analyzed: EGFR amplification, TERT promoter mutation, CDKN2A/B homozygous loss, chromosome 7 + /10- aneuploidy, MGMT methylation, IDH mutation, ATRX loss, p53 mutation and 1p19q codeletion. Subsequently, we calculated the heme biosynthesis mRNA expression signature for correlation with distinct molecular glioma markers/molecular subgroups and performed survival analyses.

**Results:**

A total of 649 patients with available data on up-to-date molecular markers and heme biosynthesis mRNA expression were included. According to analysis of individual molecular markers, we found a significantly higher heme biosynthesis mRNA expression signature in gliomas with IDH wildtype (*p* < 0.0005), without 1p19q codeletion (*p* < 0.0005), with homozygous CDKN2A/B loss (*p* < 0.0005) and with EGFR amplification (*p* = *0.001*). Furthermore, we observed that the heme biosynthesis mRNA expression signature increased with molecular subgroup aggressiveness (*p* < *0.0005*), being lowest in WHO grade 2 oligodendrogliomas and highest in WHO grade 4 glioblastomas. Finally, the heme biosynthesis mRNA expression signature was a statistically significant survival predictor after multivariate correction for all molecular markers (*p* < 0.0005).

**Conclusion:**

Our data demonstrate a significant correlation between heme biosynthesis regulation and diagnostic molecular markers and a prognostic relevance independent of these established markers. Consequently, heme biosynthesis expression is a promising biomarker for glioma aggressiveness and might constitute a potential target for novel therapeutic approaches.

## Introduction

Diffusely infiltrating gliomas are the most frequent brain tumor entity in adult patients suffering from primary central nervous system (CNS) tumors (Ohgaki and Kleihues, 2005; Ostrom et al., 2017). Whenever possible, the initial treatment of choice is maximum safe tumor resection with preservation of neurological function (Marko et al., 2014; Hervey-Jumper and Berger, 2016; Bush et al., 2017). Primarily, gliomas were classified based on specific histopathological criteria defined by the World Health Organization (WHO) in different tumor grades (WHO grades II-IV) and subtypes (astrocytoma, oligodendroglioma and oligoastrocytoma) (Louis et al., 2007a). The initiation of postoperative treatment with radio- and/or chemotherapy was mainly based on the WHO glioma grade/subtype (Schiff et al., 2007; Kumthekar et al., 2015; Taal et al., 2015). Aside from this histopathological tumor diagnosis, further important factors have been suggested to influence biology of gliomas (Engh, 2011; Cohen and Colman, 2015; Aoki et al., 2018).

Recently, we investigated the role of the heme biosynthesis pathway in the biology of diffusely infiltrating gliomas (Mischkulnig et al., 2020, 2021). Besides its well-known role as blood oxygen carrier, heme is also crucial for various critical cellular processes with important implications on tumor biology such as oxygen transportation, energy production as part of the electron transport chain and drug metabolism (Heinemann et al., 2008; Shimizu et al., 2019). In a first study, we demonstrated that significant alterations in heme biosynthesis mRNA expression compared to normal brain tissue continuously increase with the WHO tumor grade (Mischkulnig et al., 2020). In a following study, we showed that a mRNA expression signature based on 11 specific heme biosynthesis proteins is associated with survival in glioma patients (Mischkulnig et al., 2021).

Nowadays, molecular markers gained major importance in the glioma diagnosis as well as treatment decisions and are thus indispensable for optimal management of patients suffering from gliomas (Louis et al., 2016; Aoki et al., 2018; Tanase et al., 2022). In this sense, specific molecular markers such as Isocitrate-Dehydrogenase (IDH) mutations, loss of transcriptional regulator Alpha-thalassemia mental retardation X-linked protein (ATRX) and codeletions of chromosomes 1p/19q have been routinely applied for optimized glioma characterization since 2016 (Louis et al., 2007a,b, 2016). In the recently published 5*^th^* edition of the WHO classification of CNS tumors, additional molecular markers for glioma diagnostics were introduced (Louis et al., 2021; Rushing, 2021; World Health Organization [WHO], 2022). For example, mutations in the epidermal growth factor receptor (EGFR), chromosome 7 gain and chromosome 10 loss (7 +/10-) or telomerase reverse transcriptase (TERT) mutations in IDH-wild-type astrocytomas result in an “upgrading” of histopathologically classified WHO grade 2/3 without IDH mutation to “IDH-wild-type glioblastoma WHO grade 4” (Brat et al., 2018; Louis et al., 2021; Rushing, 2021). Similarly, homozygous loss of cyclin dependent kinase inhibitor (CDKN) 2A/2B results in the upgrading of lower-grade IDH-mutant gliomas to WHO grade 4 (Brat et al., 2020). In WHO grade 2 and 3 gliomas, detection of mutations in the tumor protein 53 (Tp53) gene or ATRX loss suffice for astrocytoma diagnosis, making 1p19q codeletion analysis non-obligatory in these cases (Louis et al., 2018, 2021). At present, no systematical data on the interaction of the heme biosynthesis pathway with the molecular glioma diagnosis algorithm proposed by the new WHO classification are available in the literature. This would, however, constitute an important investigation not only for an improved understanding of glioma biology but also to facilitate diagnostic and therapeutic innovation in glioma patients. Furthermore, the addition of molecular markers to routine glioma diagnosis is expected to improve its prognostic accuracy and it remains thus unclear whether the prognostic impact of heme biosynthesis expression previously demonstrated for histologically classified gliomas is still present.

The aim of this study was thus to examine the heme biosynthesis pathway expression according to relevant molecular markers in a large cohort of diffusely infiltrating gliomas and establish whether the previously established prognostic impact of heme biosynthesis expression remains present after consideration of the molecular glioma markers recently introduced into routine diagnostic work-up. To this end, we updated the tumor diagnosis of our initial “The Cancer Genome Atlas” (TCGA) glioma dataset (Cancer Genome Atlas Research Network, 2008; Brennan et al., 2013; Cancer Genome Atlas Research Network Brat et al., 2015) with relevant molecular markers based on the recently published 5*^th^* edition of the WHO classification reflecting the most recent advances in glioma characterization (Brat et al., 2018, 2020; Louis et al., 2018, 2021; Rushing, 2021). In the process, we correlated the mRNA expression signature with distinct molecular glioma markers as well as the resulting molecular subgroups. Subsequently, we investigated if the heme biosynthesis mRNA signature retains a survival impact beyond the one associated with the established molecular glioma markers.

## Materials and methods

In the present study, we conducted a systematic analysis of the impact of relevant molecular glioma markers published in the 5*^th^* edition of the WHO classification on the recently described heme biosynthesis mRNA expression signature (Mischkulnig et al., 2020, 2021; World Health Organization [WHO], 2022). This investigation was structured into three distinct analytic steps, including analyses of heme biosynthesis expression according to distinct molecular glioma markers as well as resulting molecular glioma subgroups and an investigation of functional protein-protein interactions between heme biosynthesis proteins and established molecular glioma markers. Finally, the overall survival impact of the heme biosynthesis mRNA signature was investigated after correction for distinct molecular markers status as well as the combined set of all relevant molecular markers. The investigation of the role of heme biosynthesis factors and molecular glioma markers was approved by the ethics committee of the Medical University Vienna (EK 419/2008 – amendment).

### Data collection and processing

This study included data of the Cancer Genome Atlas (TCGA) provided by the National Cancer Institute and Human Genome Research Institute as well as classification of molecular profiling data generated by the TCGA Research Network within a study on molecular profiling in diffuse gliomas published in 2016 (Cancer Genome Atlas Research Network, 2008; Brennan et al., 2013; Cancer Genome Atlas Research Network Brat et al., 2015; Ceccarelli et al., 2016). Gene expression data and information contained in the TCGA lower grade glioma and glioblastoma (GBMLGG) dataset were accessed through the Xena tool provided by University of California Santa Cruz on February 21*^st^* 2021 (The Cancer Genome Atlas [TCGA], 2016, 2018, 2019; Goldman et al., 2019). Furthermore, information on molecular markers as characterized by Ceccarelli et al. were obtained through the cBio portal on February 26*^th^* 2021 (Cerami et al., 2012; Gao et al., 2013; Ceccarelli et al., 2016). Both datasets were merged according to the unique Sample ID variable and only samples with available gene expression data as well information on molecular markers were considered for further analyses.

### Heme biosynthesis mRNA expression signature

As parameter that integrates all 11 heme biosynthesis factors responsible for metabolizing the widely used fluorescent dye 5-aminolevulinic acid (5-ALA) for intraoperative tumor visualization to the actively fluorescing metabolite protoporphyrin IX (PpIX), the heme biosynthesis mRNA expression signature was applied as described earlier (Mischkulnig et al., 2020). Factors in the heme biosynthesis mRNA expression signature include Solute Carrier Family 15 Members 1&2 (SLC15A1 & SLC15A2), Aminolevulinate-Dehydratase (ALAD), Hydroxymethylbilane-Synthase (HMBS), Uroporphyrinogen-III-Synthase (UROS), Uroporphyrinogen-Decarboxylase (UROD), Coproporphyrinogen-Oxidase (CPOX), Protoporphyrinogen-Oxidase (PPOX), ATP-binding Cassette Subfamily B Member 6 (ABCB6), ATP-binding Cassette Subfamily G Member 2 (ABCG2) and Ferrochelatase (FECH). The exact formula used to calculate this signature according to log-transformed normalized mRNA expression data in log2 (norm_value + 1) format that constitutes the linear function most accurately discriminating between typically fluorescent and non-fluorescent gliomas as previously demonstrated (Mischkulnig et al., 2020) is provided below:

mRNA expression signature

$$\;=0.107^{*}\,\text{Log2}\,{\left(\text{norm}\,\_\,\text{value}+1\right)}^{S\,L\,C\,15\,A\,1}$$

$$\;\;-1.255^{*}\,\text{Log2}\,{\left(\text{norm}\,\_\,\text{value}+1\right)}^{S\,L\,C\,15\,A\,2}$$

$$\;\;-2.223^{*}\,\text{Log2}\,{\left(\text{norm}\,\_\,\text{value}+1\right)}^{A\,L\,A\,D}$$

$$\;\;+2.634^{*}\,\text{Log2}\,{\left(\text{norm}\,\_\,\text{value}+1\right)}^{H\,M\,B\,S}$$

$$\;\;-2.928^{*}\,\text{Log2}\,{\left(\text{norm}\,\_\,\text{value}+1\right)}^{U\,R\,O\,S}$$

$$\;\;+2.988^{*}\,\text{Log2}\,{\left(\text{norm}\,\_\,\text{value}+1\right)}^{U\,R\,O\,D}$$

$$\;\;-1.428^{*}\,\text{Log2}\,{\left(\text{norm}\,\_\,\text{value}+1\right)}^{A\,B\,C\,B\,6}$$

$$\;\;+0.659^{*}\,\text{Log2}\,{\left(\text{norm}\,\_\,\text{value}+1\right)}^{C\,P\,O\,X}$$

$$\;\;-2.863^{*}\,\text{Log2}\,{\left(\text{norm}\,\_\,\text{value}+1\right)}^{P\,P\,O\,X}$$

$$\;\;-0.476^{*}\,\text{Log2}\,{\left(\text{norm}\,\_\,\text{value}+1\right)}^{A\,B\,C\,G\,2}$$

$$\;\;+2.488^{*}\,\text{Log2}\,{\left(\text{norm}\,\_\,\text{value}+1\right)}^{F\,E\,C\,H}$$

### Heme biosynthesis expression according to distinct molecular glioma markers

In a first step, heme biosynthesis expression was investigated according to distinct molecular glioma markers as outlined in the recently published 5*^th^* edition of the WHO glioma classification (World Health Organization [WHO], 2022). Markers in this investigation included presence of IDH1 mutations, 1p19q codeletions, TERT promotor mutations, ATRX loss, homozygous CDKN2A/B loss, concurrent whole chromosome 7 gain/10 loss as well as TP53 and MGMT mutational status (World Health Organization [WHO], 2022). Furthermore, EGFR amplification status and presence of homozygous CDKN2A/B loss was investigated according to the copy number variation (CNV) data available within the GBMLGG dataset. While EGFR amplification was assumed in tumors with copy number values higher than 0.6 on a log2-scale as described earlier (Stichel et al., 2018), loss of two CDKN2A or two CDKN2B copies was classified as homozygous loss.

### Heme biosynthesis expression according to molecular glioma subgroups

**FIGURE 1:**
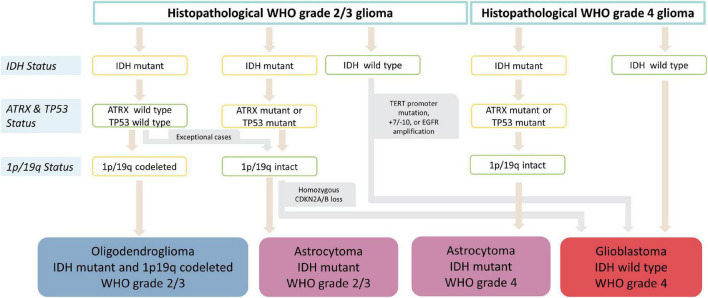
Flowchart of molecular glioma subgroup categorization. After initial histopathological tumor grading, subtyping into IDH mutant and 1p19q codeleted oligodendroglioma, IDH mutant astrocytoma, IDH wild type astrocytoma and IDH wild type glioblastoma is performed according to IDH, ATRX and 1p/19q status. Furthermore, homozygous CDKN2A/B loss in IDH mutant astrocytomas as well as presence of either TERT promoter mutation, 7+/10- aneuploidy or EGFR amplification IDH in wild type gliomas results in upgrading to WHO grade 4 independently of histopathological grading.

**FIGURE 2:**
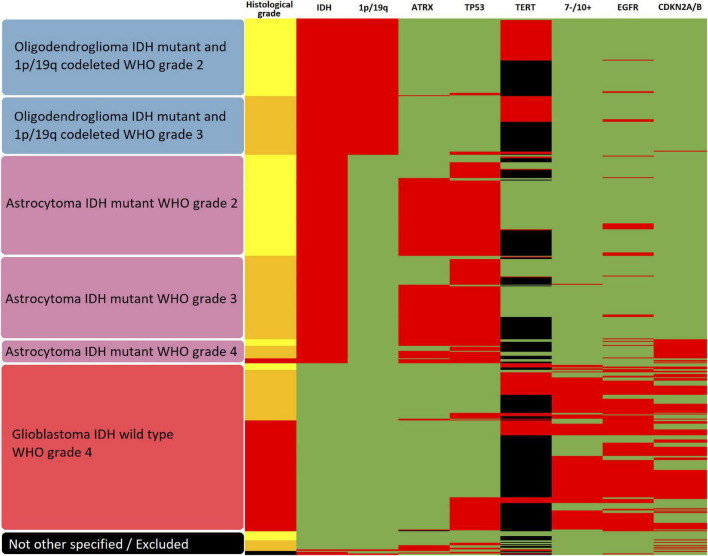
Heatmap of subgroups according to molecular glioma markers. The resulting molecular glioma subgroup as well as histological tumor grade and molecular marker status for IDH mutation, 1p/19q codeletion, ATRX loss, TP53 mutation, TERT promoter methylation, 7-/10 + aneuploidy, EGFR amplification and homozygous CDKN2A/B loss is shown for each analyzed tumor. In regard to histological grade, yellow represents WHO grade 2, orange WHO grade 3 and red WHO grade 4. For all molecular markers, green represents the unaltered status whereas red shows the presence of IDH mutation, 1p/19q codeletion, ATRX loss, TP53 mutation, TERT promoter methylation, 7-/10+ aneuploidy, EGFR amplification and homozygous CDKN2A/B loss, respectively. Missing data is represented in black.

In a second step, a glioma classification according to the status of multiple individual markers as outlined in the current 5*^th^* edition of the WHO classification of CNS tumors (World Health Organization [WHO], 2022) was performed and the heme biosynthesis mRNA expression signature was then analyzed according to these molecular glioma subgroups. The variables determining the molecular subgroups included histopathological tumor grade, IDH mutational status, 1p/19q codeletion status, ATRX loss status, EGFR amplification status, TERT promoter mutation status, presence of homozygous CDKN2A/B loss, Tp53 mutation status and 7 + /10- aneuploidy status. Subtyping was performed by a trained neuropathologist (T.R.). The schematic algorithm of subtyping is shown in Figure 1 and a comprehensive overview of all observed combinations of markers with resulting glioma subgroups is provided in Figure 2.

### Protein-protein interaction analysis

In a third step, an investigation of potential direct metabolic interactions between heme biosynthesis factors and molecular glioma markers was performed using the publicly available STRING online database system^1^ (Szklarczyk et al., 2019). To analyze interactions between all proteins examined in this study, the following input was entered: IDH, ATRX, MGMT, EGFR, TERT, CDKN2A, CDKN2B, P53, SLC15A1, SLC15A2, ALAD, HMBS, UROS, UROD, CPOX, PPOX, ABCB6, ABCG2, FECH.

### Survival analysis

In order to investigate the previously demonstrated prognostic effect of the heme biosynthesis mRNA expression signature after correction for molecular marker status, the overall survival data included in the TCGA GBMLGG dataset were examined. The association between mRNA expression signature and overall patient survival was examined after correction for distinct molecular markers (univariate analysis) as well as after correction for the complete set of all nine investigated molecular markers (multivariate analysis).

### Statistical analysis

Statistical investigations were performed using SPSS statistical software (Version 27.0, SPSS Inc.). Descriptive analyses included patient age, gender, histopathological tumor subtypes and WHO grades as well as the mRNA expression signature and the molecular marker status for the entire cohort and subgroups according to IDH status. In the initial step, where the mRNA expression signature was visualized according to separate molecular markers, presence of a difference between the groups was tested using unpaired *t*-tests. To control for multiple testing, markers with significant results in this univariate analysis where then included in a multivariate linear regression model, and the p-values derived from this model were finally used to detect differences according to distinct markers. In the second step, distinct molecular subgroups according to the WHO classification were defined and mRNA expression signature was investigated between these subgroups using ANOVA with *post hoc* Fisher’s least significant difference (LSD) test between distinct subgroups. For the survival analysis, Cox regression models including the metric mRNA expression signature variable alone or in in combination with either one distinct molecular marker or the complete set of all nine molecular markers were created. Molecular markers were used as categorical variables (normal/altered/missing). While inferential statistical analysis was performed using the metric heme biosynthesis mRNA expression signature variable, patients were categorized into three equally sized mRNA expression signature subgroups (“low”, “intermediate” and “high”) for survival visualization in Kaplan-Meier curves. Multiple testing was addressed by using a multivariate regression model and ANOVA with *post hoc* Fisher’s LSD ensuring that type I error for each main hypothesis remained at 5% and thus the resulting p-values were considered statistically significant under the commonly used threshold of *p* = 0.05.

## Results

From altogether 1122 patients with available data on the molecular marker status, sequencing data were available for 649 patients that therefore formed our study cohort. An IDH mutation was present in a majority of 423 (65.2%) of patients as compared to 226 (34.8%) patients with IDH wildtype tumors. The median patient age of the entire cohort was 47 years (14–89 years) with a male: female ratio of 1.36:1. Further details on the patient characteristics of the entire study cohort as well as IDH mutant and IDH wildtype subgroups are provided in Table 1.

**TABLE 1:** Patient characteristics.

		Overall		IDH mutant		IDH wildtype	
		*n*	(%)	*n*	(%)	*n*	(%)
**Number of patients**		**649**	**100**	423	65.2	226	34.8
**Age**	**median (range)**	**47 (14-89)**		41 (14-75)		58 (21-89)	
**Gender**	**(male : female)**	**1.36**		1.26		1.59	
**Hisological grade**							
	**WHO grade II**	**243**	**(37.4)**	224	(53.0)	19	(8.4)
	**WHO grade III**	**261**	**(40.2)**	189	(44.7)	72	(31.9)
	**WHO grade IV**	**140**	**(21.6)**	6	(1.4)	134.00	(59.3)
	**N/A**	**5**	**(0.8)**	4	(0.9)	1	(0.4)
**1p19q**							
	**Codeletion**	**169**	**(26.0)**	169	(40.0)	0	-
	**No Codeletion**	**480**	**(74.0)**	254	(60.0)	226	(100.0)
**ATRX status**							
	**No loss**	**454**	**(70.0)**	237	(56.0)	217	(96.0)
	**Loss**	**194**	**(29.9)**	186	(44.0)	8	(3.5)
	**N/A**	**1**	**(0.2)**	0	-	1	(0.5)
**Tp53 mutation**							
	**Mutated**	**294**	**(45.3)**	241	(57.0)	53	(23.5)
	**Not mutated**	**355**	**(54.7)**	182	(43.0)	173	(76.5)
**CDKN2A/B homozygous loss**							
	**Homozygous loss**	**145**	**(22.3)**	27	(6.4)	118	(52.2)
	**No homozygous loss**	**504**	**(77.7)**	396	(93.6)	108	(47.8)
**TERT promoter mutation**							
	**Mutated**	**108**	**(16.6)**	92	(21.7)	16	(7.1)
	**Not mutated**	**207**	**(31.9)**	145	(34.3)	62	(27.4)
	**N/A**	**334**	**(51.5)**	186	(44.0)	148	(65.5)
**+7/-10q**							
	**Aneuploidy**	**149**	**(23.0)**	1	(0.2)	148	(65.5)
	**No aneuploidy**	**500**	**(77.0)**	422	(99.8)	78	(34.5)
**EGFR amplification**							
	**Amplification**	**186**	**(28.7)**	28	(6.6)	158	(69.9)
	**No Amplification**	**463**	**(71.3)**	395	(94.4)	68	(30.1)
**MGMT promoter methylation**							
	**Methylated**	**467**	**(72.0)**	392	(92.7)	75	(32.2)
	**Unmethylated**	**154**	**(23.7)**	31	(7.3)	123	(54.4)
	**N/A**	**28**	**(4.3)**	0	-	28	(12.4)

The bold formatting represents the overall cohort in contrast to the subcohorts of IDHwt and IDHmutant gliomas.

### Analysis of individual molecular markers in the study cohort

With regard to the investigated molecular markers, IDH mutations were found in 423 (65.2%) patients and 1p19q codeletion was detected in 169 (26.0%) patients. Further, ATRX loss was present in 194 (29.9%) of 648 cases, whereas no ATRX status was available in 1 case. Moreover, Tp53 mutations were observed in 294 (45.3%) patients. Furthermore, 7 + /10- aneuploidy was present in 149 (23.0%) patients and EGFR amplification was observed in 186 (28.7%) cases. Finally, MGMT promoter methylation was detected in 467 (75.2%) of 621 patients, whereas the methylation status was missing in the remaining 28 cases. An overview of the frequency of molecular marker expression in the entire cohort as well as subgroups according to IDH mutation status is provided in Table 1.

### Differences in mRNA expression signature according to individual molecular markers

In the initial univariate analysis, significantly higher mRNA expression signature were observed in tumors with IDH mutations (-19.99 ± 3.85 vs. -26.33 ± 2.84; *p* < 0.0005), without 1p19q codeletion (-22.88 ± 4.21 vs. -27.66 ± 2.30; *p* < 0.0005), ATRX wildtype (-23.58 ± 4.83 vs. -25.45 ± 2.84; *p* < 0.0005), 7 + /10- aneuploidy (-19.46 ± 3.2 vs. 25.52 ± 3.71; *p* < 0.0005), EGFR receptor amplification (-20.15 ± 3.86 vs. -25.72 ± 3.54; *p* < 0.0005), homozygous CDKN2A/B loss (-20.19 ± 3.61 vs. -25.26 ± 3.96; *p* < 0.0005) and unmethylated MGMT promoter status (-21.32 ± 4.39 vs. 25.39 ± 3.78; *p* < 0.0005). In contrast, no significant differences in the mRNA expression signature were observed according to Tp53 mutational status (-24.32 ± 3.86 vs. -23.97 ± 4.82; *p* = *0.301*) and TERT mutational status (-24.46 ± 4.53 vs. -25.23 ± 2.87; *p* = *0.074*).

**FIGURE 3:**
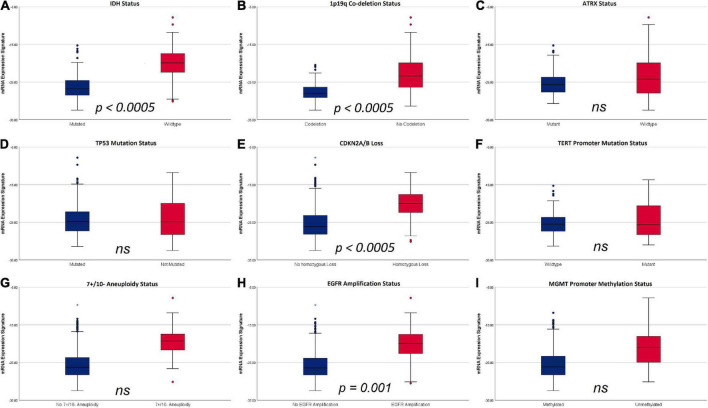
Boxplots of mRNA expression signature distribution according to distinct molecular glioma markers. **(A)** IDH status: IDH wild type tumors showed a significantly higher mRNA expression signature compared to IDH mutant tumors. **(B)** 1p19q codeletion status: tumors without codeletion showed a significantly higher mRNA expression signature compared to codeleted tumors. **(C)** ATRX status: No significant differences were observed according to ATRX status. **(D)** TP53 mutation status: No significant differences were observed according to TP53 mutation status. **(E)** Homozygous CDKN2A/B loss: tumors with homozygous CDKN2A/B loss had significantly higher mRNA expression signatures compared to tumors without homozygous CDKN2A/B loss. **(F)** TERT mutation status: No significant differences were observed according to TERT mutation status. **(G)** 7 + /10- aneuploidy status: No significant differences were observed according to 7 + /10- aneuploidy status. **(H)** EGFR amplification: mRNA expression signature were significantly higher in tumors with EGFR amplification. **(I)** MGMT promoter methylation status: No significant differences in mRNA expression signature were observed according to MGMT promoter methylation status.

The subsequent multivariate analysis was calculated for molecular markers with a significant impact on mRNA expression signature in the univariate analyses. According to these data, the multivariate regression model identified the IDH mutational status (*p* < 0.0005), 1p19q codeletion (*p* < 0.0005), homozygous CDKN2A/B loss (*p* < 0.0005) and EGFR amplification (*p* = 0.001) as independent factors influencing heme biosynthesis mRNA expression. In contrast, no independent effect was present for 7 + /10- aneuploidy (*p* = 0.069), ATRX status (*p* = 0.176) and MGMT promoter methylation (*p* = 0.733). The relation of mRNA expression signature according to individual molecular markers with p-values representing the results of the multivariate analysis are shown in Figure 3.

### Categorization of gliomas into subgroups according to molecular markers

Applying the molecular classification of gliomas, 619 (95.4%) tumors could be unambiguously assigned to a distinct molecular subgroup. Of these, 164 (26.5%) tumors were classified as IDH mutant and 1p/19q codeleted oligodendrogliomas, 252 (40.7%) tumors were classified as IDH mutant astrocytomas and the remaining 203 (32.8) tumors as IDH wildtype glioblastomas. Regarding the WHO grade, grade 2 accounted for 93 (56.7%) cases of IDH mutant and 1p/19q codeleted oligodendrogliomas and 122 (48.4%) cases of IDH mutant astrocytomas. In contrast, WHO grade 3 was present in 71 (43.4%) cases of IDH mutant and 1p/19q codeleted oligodendrogliomas and 101 (40.1%) cases of IDH mutant astrocytomas. Furthermore, WHO grade 4 was present in 29 (11.5%) cases of IDH mutant astrocytomas. In contrast, a total of 30 (4.6%) cases could not be clearly categorized due to missing information on histological WHO grade in 5 cases (0.8%) and molecular marker combinations not consistent with any specified glioma subgroup in 25 cases (3.8%) and were thus not included in the molecular subgroup analysis. It is of note, that 139 (59.9%) cases of glioblastomas and IDH mutant WHO grade 4 astrocytomas were histopathologically classified as WHO grade 4 tumors, whereas the remaining cases were histopathologically classified as grade 2 (17 cases; 7.3%) and grade 3 (76 cases; 32.8%) tumors and subsequently classified as WHO grade 4 solely due to molecular markers.

### Differences in mRNA expression signature according to molecular glioma subgroups

**FIGURE 4:**
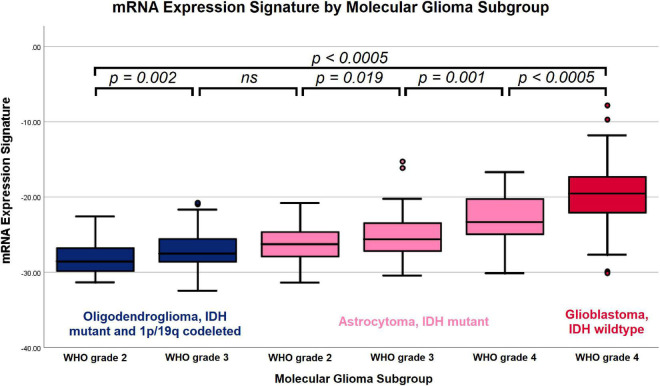
Boxplot of mRNA expression signature by molecular glioma subgroup. Increasing mRNA expression signatures were observed in IDH mutant and 1p/19q codeleted oligodendrogliomas WHO grade 2, IDH mutant and 1p/19q codeleted oligodendrogliomas WHO grade 3, IDH mutant astrocytomas WHO grade 2, IDH mutant astrocytomas WHO grade 3, IDH mutant astrocytomas WHO grade 4 and IDH wild type glioblastomas WHO grade 4. The overall differences between the subgroups and the between all individual subgroups except for oligodendrogliomas WHO grade 3 vs. IDH mutant astrocytomas WHO grade 2 were statistically significant.

The mRNA expression signature values increased with the aggressiveness of the molecular subgroups: (1) -28.28 ± 1.92 in IDH mutant and 1p/19q codeleted oligodendrogliomas WHO grade 2, (2) -26.90 + 2.43 in IDH mutant and 1p/19q codeleted oligodendrogliomas WHO grade 3, (3) -26.16 ± 2.27 in IDH mutant astrocytomas WHO grade 2, (4) -25.24 ± 2.80 in IDH mutant astrocytomas WHO grade 3, (5) -23.22 ± 3.78 in IDH mutant astrocytomas WHO grade 4 and (6) -19.55 ± 3.55 in IDH wild type glioblastomas WHO grade 4. The overall differences between these subgroups were statistically highly significant (*p* < *0.0005*), and the *post hoc* subgroup analysis demonstrated significant differences between all distinct subgroups except for oligodendrogliomas WHO grade 3 and astrocytomas WHO grade 2. The distribution of mRNA expression signature according to molecular glioma subgroups is visualized in Figure 4 and the detailed results of the *post hoc* analysis comparing pairs of distinct subgroups is provided in Table 2.

**TABLE 2:** Detailed results of ***post hoc*** subgroup analysis.

p-Values of *post hoc* Fisher’s LSD test	Oligodendroglioma, IDH mutant and 1p/19q codeleted, WHO grade 2	Oligodendroglioma, IDH mutant and 1p/19q codeleted, WHO grade 3	Astrocytoma, IDH mutant, WHO grade 2	Astrocytoma, IDH mutant, WHO grade 3	Astrocytoma, IDH mutant, WHO grade 4	Glioblastoma, IDH wild type, WHO grade 4

Oligodendroglioma, IDH mutant and 1p/19q codeleted, WHO grade 2		0.002	< 0.0005	<0.0005	< 0.0005	<0.0005
Oligodendroglioma, IDH mutant and 1p/19q codeleted, WHO grade 3	0.002		0.087	< 0.0005	<0.0005	< 0.0005
Astrocytoma, IDH mutant, WHO grade 2	< 0.0005	0.087		0.019	< 0.0005	<0.0005
Astrocytoma, IDH mutant, WHO grade 3	< 0.0005	<0.0005	0.019		0.001	< 0.0005
Astrocytoma, IDH mutant, WHO grade 4	< 0.0005	<0.0005	< 0.0005	0.001		< 0.0005
Glioblastoma, IDH wild type, WHO grade 4	< 0.0005	<0.0005	< 0.0005	<0.0005	< 0.0005	


Significant differences between the respective subgroups are highlighted in green, whereas non-significant differences are highlighted in orange. Fields were columns and rows represent the same subgroups are grayed out.

### Exploration of protein-protein interactions

**FIGURE 5:**
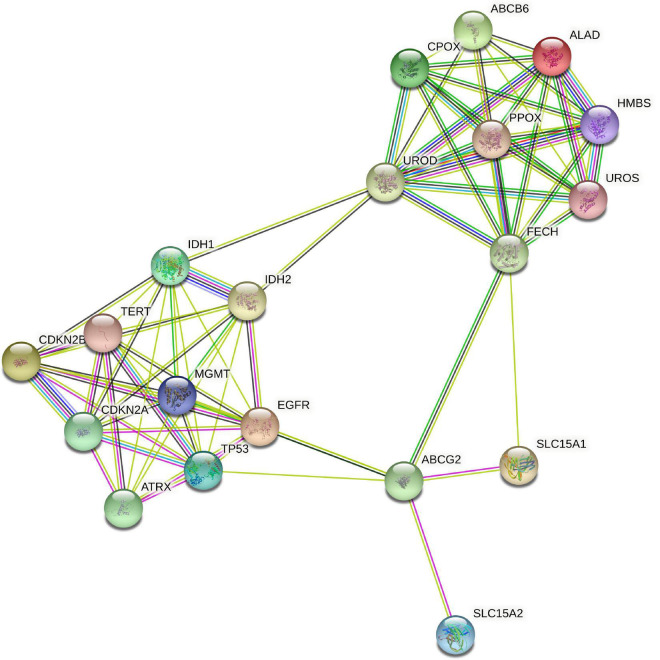
STRING protein interaction analysis of heme biosynthesis and glioma markers. STRING database analysis of metabolic interactions between heme biosynthesis factors and molecular glioma markers demonstrated that both subgroups of proteins form tightly interlinked networks that are nearly completely isolated from each other.

Next, we investigated potential connections between the proteins that form the basis for the molecular markers and proteins of the heme biosynthesis pathway. Using the STRING database, we thus mapped all heme biosynthesis enzymes and all molecular markers (apart from, e.g., 7 + /10- aneuploidy and 1p/19q codeletion where no directly corresponding protein is identifiable). The resulting interaction graph is shown in Figure 5. Molecules of the heme biosynthesis signature cluster together tightly and so do the molecular glioma markers. However, hardly any connections between these two clusters are present, indicating the presence of two functionally independent molecular systems.

### Analysis of an additional survival impact of heme biosynthesis compared to molecular markers

**FIGURE 6:**
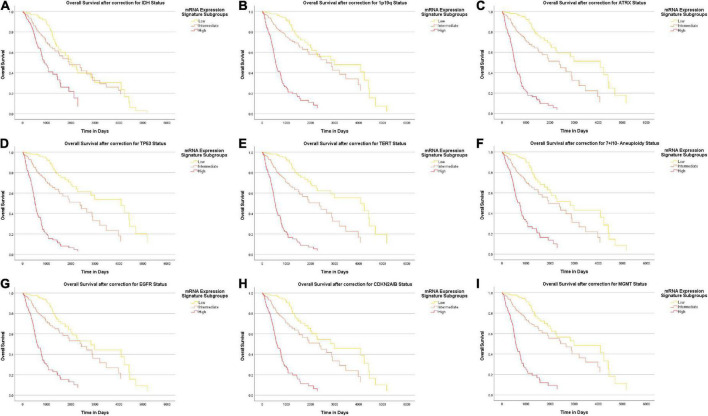
Survival-curves for overall survival after correction for distinct molecular markers. Kaplan-Meier curves for overall patient survival are shown after correction for distinct molecular markers. The heme biosynthesis mRNA expression signature showed a statistically significant correlation (*p* < 0.0005) with patient survival after correction for all investigated markers in separate analyses including IDH mutation status **(A)**, 1p19q codeletion status **(B)**, ATRX loss status **(C)**, TP53 mutation status **(D)**, TERT promoter mutation status **(E)**, 7 + /10- aneuploidy status **(F)**, EGFR amplification status **(G)**, homozygous CDKN2A/B loss status **(H)** and MGMT promotor methylation status **(I)**.

**FIGURE 7:**
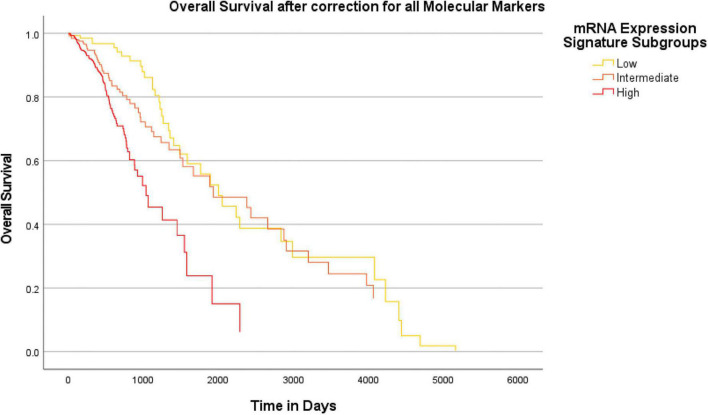
Survival-curves for overall survival after correction for all molecular markers in a combined model. Kaplan-Meier curves for overall patient survival are shown after correction for all molecular markers investigated in this study within a combined Cox regression model. The heme biosynthesis mRNA expression signature showed a statistically significant (*p* < 0.0005) correlation with patient survival even after combined correction for all molecular glioma markers.

Overall, we found a median overall survival of 1547 days in the entire cohort. With regard to the IDH mutational status, the median overall survival was markedly longer in IDH mutant gliomas (median survival 2907 days) as compared to IDH wildtype gliomas (median survival 454 days). In a subsequent analysis, we tested if the heme biosynthesis mRNA signature has an additional survival impact beyond the one associated with molecular glioma markers. For this analysis, we performed Cox-regression analyses and plotted Kaplan Meier curves after correction for the molecular marker status. After initial correction for one distinct molecular marker at a time, a highly significant survival impact of the heme biosynthesis signature could be demonstrated after correction for each investigated marker including IDH mutation (*B* = 0.095, *p* < 0.0005), ATRX loss (*B* = 0.194, *p* < 0.0005), 1p/19q codeletion (*B* = 0.191, *p* < 0.0005), TERT (*B* = 0.197, *p* < 0.0005), 7 + /10- aneuploidy (*B* = 0.145, *p* < 0.0005), EGFR amplification (*B* = 0.164, *p* < 0.0005), homozygous CDKN2A/B loss (*B* = 0.175, *p* < 0.0005), TP53 mutation (*B* = 0.209, *p* < 0.0005) and MGMT promoter methylation (*B* = 0.183, *p* < 0.0005). Kaplan-Meier survival curves stratified for mRNA expression signature subgroups after correction for distinct molecular markers are shown in Figure 6. Likewise, the heme biosynthesis mRNA expression signature remained a statistically significant survival predictor (*B* = 0.093, *p* < 0.0005) after multivariate correction for all molecular markers in a single cox-regression model. Overall survival curves after correction for all nine molecular markers are shown in Figure 7.

## Discussion

The prognosis and clinical outcome of diffusely infiltrating gliomas is very variable and thus a more profound understanding of glioma biology is urgently needed to better estimate the individual patient prognosis and guide treatment decisions (Stupp et al., 2005; Yang et al., 2013). The heme biosynthesis pathway constitutes a promising factor that has recently been linked to survival in several tumor entities including also diffusely infiltrating gliomas (Su et al., 2020; Mischkulnig et al., 2021). Heme is not only relevant in oxygen transportation, but is also involved in a number of essential cellular processes like energy production where it is important part of the electron transport chain, signal transduction and drug metabolism (Layer et al., 2010). To date, however, the precise understanding of the heme biosynthesis regulation and biology in gliomas is limited. Based on these considerations, we recently described a heme biosynthesis gene expression signature that is significantly enriched in typically fluorescent WHO grade IV gliomas (Mischkulnig et al., 2020). Furthermore, this signature was shown to correlate with glioma patient survival independently of established prognostic factors including WHO grade, sex, patient age and tumor subtype including the Verhaak subtype for glioblastomas (Mischkulnig et al., 2021). This heme biosynthesis gene expression signature constitutes a promising biomarker for aggressive glioma behavior and might thus markedly improve the routine patient management.

Traditionally, glioma classification as suggested by the WHO was mainly based on histopathology and first molecular markers including IDH mutations, ATRX loss and 1p/19q codeletion have been integrated into routine glioma subtyping since the updated WHO criteria published in 2016 (Louis et al., 2007a,b, 2016). Since neurooncology is a dynamic field and updates are regularly made based on novel research findings, the recently published 5^th^ edition of the WHO classification includes a number of additional molecular markers such as homozygous CDKN2A/B loss, TERT promoter mutation and chromosome 7 polysomy with chromosome 10 monosomy in the routine glioma diagnosis (Louis et al., 2021). Consequently, the role of molecular markers gained practice-changing importance in routine glioma diagnosis nowadays to enable precise tumor characterization (Louis et al., 2021). A further novel characteristic feature of the current WHO classification is that the diagnosis of “glioblastoma” will be reserved to IDH wildtype tumors, whereas IDH mutant WHO grade 4 lesions will be classified as “WHO grade 4 astrocytoma” (Louis et al., 2021). The aim of this study was to take these recent advances in diagnostic workup as part of the current and new standard in diagnosis of diffusely infiltrating gliomas into account and investigate whether heme biosynthesis expression retains prognostic relevance after this substantial update of glioma classification. We therefore included the complete set of established and novel molecular markers into the present study.

### Association of the heme biosynthesis mRNA expression signature with individual molecular markers and resulting molecular glioma subgroups

According to the analysis in regard to distinct molecular markers, we found that the mRNA expression signature was significantly elevated in IDH wildtype tumors, cases without 1p/19q codeletion, gliomas with homozygous loss of CDKN2A/B and in the EGFR amplification group. Therefore, we observed higher heme biosynthesis mRNA signatures in tumors with a molecular marker profile associated with more aggressive glioma behavior. To our knowledge, this is the first study demonstrating the significant relation of these specific molecular markers with the heme biosynthesis mRNA expression signature in a large cohort of diffusely infiltrating gliomas whereas two previous studies described higher mRNA expression signatures in more aggressive histopathological gliomas subtypes and grades (Mischkulnig et al., 2020, 2021).

According to the subsequent analysis of resulting molecular glioma subgroups, we found increasing values of the heme biosynthesis mRNA expression signature in these molecular subgroups from IDH mutant and 1p/19q codeleted oligodendrogliomas WHO grade 2 to IDH mutant and 1p/19q codeleted oligodendrogliomas WHO grade 3, IDH mutant astrocytomas WHO grade 2, IDH mutant astrocytomas WHO grade 3, IDH mutant astrocytomas WHO grade 4 and IDH wild type glioblastomas WHO grade 4. To our knowledge, this is the first report that demonstrates a significant correlation of heme biosynthesis mRNA expression signature with molecular subgroups according to the new WHO classification in a large cohort of DIG (World Health Organization [WHO], 2022). Interestingly, our findings demonstrate that heme biosynthesis mRNA expression signature is not only associated with the WHO glioma grade (Mischkulnig et al., 2020), but also with the molecular glioma subgroups defined by the new WHO classification (World Health Organization [WHO], 2022).

### Analysis of potential protein-protein interactions

Next, we explored potential metabolic interactions between heme biosynthesis enzymes/transporters and molecular glioma markers on protein level using the STRING database. This analysis depicts known and predicted interactions between proteins. It revealed that the heme biosynthesis factors and the molecular glioma markers each form tightly interlinked networks. However, they seem to be nearly completely isolated from one another. Thus, even though the molecular markers and the heme biosynthesis signature are each associated with aggressive phenotypes and oftentimes correlate with each other, they apparently represent two functionally distinct systems. This is a crucial observation as it indicates that the heme biosynthesis pathway might constitute a therapeutic target and biomarker independent of the other molecular markers already implicated in glioma diagnosis so far.

### Survival analysis

Finally, we performed survival analyses to provide a complete picture of the relation between heme biosynthesis regulation and the newly introduced molecular glioma markers. To this end, the overall survival impact of the heme biosynthesis mRNA signature was demonstrated after correction for distinct molecular markers as well as the combined set of all relevant molecular markers. It is important to note that in line with our previous analysis, patients showing a heme biosynthesis pattern associated with typically 5-ALA fluorescent tumors showed shorter overall survival times (Mischkulnig et al., 2020, 2021). This is well in accordance with clinical observations that demonstrated low-grade gliomas with detectable 5-ALA fluorescence to show poorer survival times than histopathologically comparable non-fluorescent gliomas (Jaber et al., 2019; Hosmann et al., 2021). Since 5-ALA guidance evidently results in improved extents of resection in glioma surgery which are in turn associated with improved patient survival, the observed survival differences are exceedingly unlikely be caused by resulting intraoperative fluorescence patterns but suggest heme biosynthesis expression as a functional factor in glioma cell behavior (Stummer et al., 2006; Mirza and Shamim, 2017). These observations further substantiate that heme biosynthesis is a factor driving aggressive glioma phenotypes and has additional prognostic significance compared to the established molecular glioma markers.

### Implications for the association between heme biosynthesis expression on mRNA and protein level

Another crucial point that needs to be addressed in the interpretation of this study based on mRNA data are the recent investigations that found mRNA levels of certain heme biosynthesis factors to not directly correlate with expression on protein level (Pustogarov et al., 2017; Mischkulnig et al., 2022). While an initial study by Pustogarov et al. demonstrated an indirect correlation between CPOX mRNA and protein levels, our more recent analysis of intramitochondrial heme biosynthesis factors found no correlation for CPOX, PPOX and FECH whereas a direct correlation was only present for ABCG2 (Pustogarov et al., 2017; Mischkulnig et al., 2022). While a direct and linear association between mRNA and protein levels seems therefore to be absent for at least some heme biosynthesis factors, the consistent tendency of higher heme biosynthesis mRNA expression signature values as found in typically fluorescent gliomas in the respective more aggressive glioma phenotype across various analyses makes a mere coincidence in the sense of repeated alpha errors exceedingly unlikely (Mischkulnig et al., 2020, 2021). Furthermore, there is increasing evidence that the presence of clinically observable 5-ALA induced fluorescence is also associated with poor patient survival (Jaber et al., 2019; Hosmann et al., 2021). Since tumor visualization relies on the metabolization of non-fluorescent 5-ALA to Protoporphyrin IX within the heme biosynthesis pathway, an association between metabolically active heme biosynthesis protein levels and patient survival seems likely (Pustogarov et al., 2017; Harada et al., 2022; Mischkulnig et al., 2022). Since prognostic effects can therefore be established for the mRNA expression signature as well as 5-ALA induced fluorescence that is highly likely to be facilitated by heme biosynthesis upregulation on protein level, the presence of a complex association between both expression levels seems plausible.

### Relevance for future biological and clinical research

Altogether, the results of our present study including a large cohort of TCGA patients with DIG contribute further evidence that the heme biosynthesis pathway plays a crucial role in glioma biology and aggressiveness. Most notably, the findings of this study provide first evidence that heme biosynthesis expression retains prognostic relevance after correction for the molecular markers recently introduced in the routine diagnostic classification of gliomas. Therefore, the heme biosynthesis mRNA expression signature could open up a novel area of drug development targets. Since heme biosynthesis upregulation apparently represents a particularly aggressive and thus malignant phenotype, it seems advisable to target enzymes of that pathway for therapeutic purposes. Importantly, the protein interaction analysis and the survival analysis indicate that heme biosynthesis is an independent molecular target on its own, rather than a mere epiphenomenon of established molecular markers. Thus, future research should aim at altering proteins of that pathway. Besides possibilities of highly targeted interventions via small molecule inhibitors or other means of gene knockdown like RNA interference, the already clinically approved tyrosine kinase inhibitor lapatinib has recently been shown to alter heme metabolism and thus constitutes a promising candidate for further research (Smyth et al., 2019; Howley et al., 2020; Mansi et al., 2022). While extensive cell and molecular biology studies will be necessary to investigate the benefit of such therapies in glioma patients, the research presented in this study provides an important first step to guide heme biosynthesis-based therapeutic approaches. Furthermore, heme biosynthesis expression together with other newly established glioma biomarkers such as long non-coding RNA expression, thrombospondin-1 and circulating biomarker panels may in future assist to perform even more precise prognostic assessments of newly diagnosed gliomas (Tanase et al., 2015, 2022; Pop et al., 2018).

### Limitations

The following limitations should be considered in the interpretation of this study (Ostrom et al., 2017): The presented investigation is constituting a retrospective analysis of a large cohort of TCGA glioma patients. While the present study is crucial in providing evidence that the heme biosynthesis pathway impacts glioma biology and prognosis beyond the effect of the currently established molecular glioma markers and is thus crucial to highlight the continued importance of this field of research, future studies are clearly warranted. In this sense, a comprehensive prospective analysis of protein and mRNA level expression levels as well as intraoperative 5-ALA status and patient survival in a sufficiently sized cohort should be performed in order to ultimately establish the role of heme biosynthesis regulation as prognostic glioma marker (Ohgaki and Kleihues, 2005). The molecular glioma classification performed in this study was based on the recently updated WHO classification of CNS tumors. Thus, the markers examined are still novel and may not be fully established in all laboratories worldwide at the current time point.

## Conclusion

In the present study, we investigated the association of the heme biosynthesis expression and relevant individual molecular markers as well as molecular subgroups according to the newest WHO classification. According to our data, we found a clear correlation for most analyzed individual molecular markers with the heme biosynthesis expression. Furthermore, we observed that heme biosynthesis expression increased with the aggressiveness of the molecular subgroups. Moreover, protein interaction analysis indicated that there is no relevant functional interaction between the heme biosynthesis pathway and the analyzed molecular glioma markers. Finally, we found that the heme biosynthesis expression has an additional survival impact compared to the established molecular glioma markers and the previously demonstrated prognostic relevance remains present after correction for these routine diagnostic markers introduced by the most recent WHO classification. Therefore, heme biosynthesis remains a promising independent biomarker for glioma aggressiveness and potential target for future development of novel therapeutic approaches.

## Data availability statement

Publicly available datasets were analyzed in this study. This data can be found here: TCGA – The Cancer Genome Atlas.

## Ethics statement

The investigation of the role of heme biosynthesis factors and molecular glioma markers was approved by the Ethics Committee of the Medical University Vienna (EK 419/2008 – amendment). Written informed consent from the [patients/participants OR patients/participants legal guardian/next of kin] was not required to participate in this study in accordance with the national legislation and the institutional requirements.

## Author contributions

GW, FE, and MrM performed conception and design of the study, BK, TR-P, MB, AL, MtM, LW, SH-J, JP, and MSB contributed to the conception and design of the study. MrM managed the database. MrM, FE, and MB performed the statistical analysis. FE and MrM wrote the first draft of the manuscript. GW and JP finalized the manuscript. All authors contributed to manuscript revision, read, and approved the submitted version.

## Footnotes

1 https://string-db.org (Version 11.0).
